# Psychological experiences and needs of perinatal women experiencing intimate partner violence: a qualitative meta-synthesis

**DOI:** 10.3389/fpubh.2025.1678360

**Published:** 2025-10-16

**Authors:** Ruifang Wen, Shanshan Wang, Kangya Yang, Hongyan Xu

**Affiliations:** Department of nursing, Women’s Hospital School of Medicine, Zhejiang University, Hangzhou, Zhejiang, China

**Keywords:** intimate relationship violence, perinatal period, experience, demand, qualitative meta-synthesis

## Abstract

**Background:**

More than a quarter of women aged 15 to 49 who have had a partner worldwide have experienced varying degrees of intimate partner violence (IPV). Cultural differences lead to different perceptions of intimate relationship violence among women, as well as varying degrees and forms of intimate relationship violence they experience.

**Aim:**

This study aimed to systematically evaluate the psychological experiences and needs of women who experienced intimate relationship violence during the perinatal period, helping clinical nursing staff identify manifestations of violence and provide targeted assistance.

**Methods:**

We systematically searched PubMed, Cochrane Library, Web of Science, CINAHL, PsycINFO, Embase, CNKI, Wanfang Database, VIP Database, China Biomedical Literature Service System to involve relevant literature on the experience and needs of intimate relationship violence among perinatal women. The search period was from the establishment of the database to July 2025. An initial search using the keywords “intimate partner violence,” “pregnancy,” “perinatal,” and “qualitative research” retrieved 2,980 articles. We used JBI quality evaluation criteria to assess the quality of the included studies. This study followed the Enhancing transparency in reporting the synthesis of qualitative research (ENTREQ) guidelines.

**Results:**

A total of 16 studies were included. Four themes and nine sub themes were summarized and synthesized: Theme 1: Negative experiences (① aggravated physical discomfort symptoms, ② severe psychological trauma); Theme 2: Poor of maternal role adaptation (① Weakening of the bond with children, ② Lack of confidence in parenting), Theme 3: Resilience from motherhood (① Self-regulation, ② Seeking change); Theme 4: Neglected needs (① Information needs, ② Social support needs).

**Conclusion:**

Perinatal women are prone to various forms of violence. This can lead to severe physical and psychological trauma as well as adverse pregnancy outcomes. Medical healthcare personnel should be trained to identify violent behaviors. Appropriate communication skills should be employed to expose intimate relationship violence. Training in skills such as parenting and psychological counseling should be provided to perinatal women. Social support organizations should offer economic, policy, legal and psychological assistance to perinatal women.

## 1 Introduction

Intimate relationship violence (IPV) refers to physical, sexual, or psychological harm inflicted by an intimate partner. It includes physical assault, sexual coercion, psychological abuse and controlling behavior (1). According to the World Health Organization, over a quarter (27%) of women aged 15 to 49 who have had a partner worldwide have experienced varying degrees of IPV (2). A systematic review study covering 40 + countries around the world indicates that physical violence accounts for 10%, psychological violence for 26%, sexual violence for 9%, verbal violence for 16%, and economic violence for 26%. Considering all types of violence together, the event rate for any IPV was 26% (3). IPV is a global public health issue. It seriously violates women’s human rights and has infiltrated individuals and families of different cultures, races, social classes, and economic levels around the world. It has caused serious consequences such as family instability and social conflicts (4).

WHO guidelines state the perinatal period refers to the duration of pregnancy and the year after birth (5). When women experience intimate partner violence during the perinatal period, it is referred to as perinatal intimate partner violence (6). The perinatal period is an important stage in a woman’s entire life cycle. The demands in terms of physiology, psychology, and the social support system have all changed. Therefore, experiencing IPV during the perinatal period will exacerbate the adverse consequences brought about by the violence. Research showed that 32.7% of women have experienced at least one IPV throughout the entire perinatal period (7). IPV can cause severe physical and psychological trauma and long-term sequelae for pregnant women during the perinatal period. For instance, injuries, gestational hypertension, chronic pain, gynecological diseases, pre-natal and post-natal depression, anxiety disorders, and post-traumatic stress syndrome (8–11). It also caused serious adverse pregnancy outcomes. Such as miscarriage, stillbirth, premature birth, low birth weight of newborns, and decreased rates of exclusive breastfeeding (2, 12–15). Exposure to IPV during the perinatal period can lead to some unhealthy behaviors, such as drug use, alcohol abuse, and suicidal tendencies. The offspring of women who suffered from IPV were at a higher risk of developing depression, anxiety, and other problems. This may have potential long-term effects on children’s health and social and psychological well-being (16). History of trauma was associated with a more severe clinical phenotype of perinatal depression (PND) and decreased resilience level (17).

Research showed that only half of all perinatal women who suffered from IPV choose to seek help. The majority of them obtain assistance from informal institutions such as family and friends (18). In Japan, only 6.9% of perinatal care institutions have implemented IPV screening (19). The reasons for society’s neglect of IPV are quite complex. For instance, there is the stigmatization of women who have suffered from IPV. Lack of relevant knowledge (20). Fear of revealing the consequences of domestic violence. The neglect of the importance of domestic violence by health service institutions and other departments (21). And concerns about the vulnerability of the fetus or pregnancy (22).

Many studies have shown that attention should be paid to and early prevention of perinatal IPV and intervention should be given to women who suffer from IPV, many research topics focused on epidemiological studies of perinatal IPV, such as the incidence of perinatal IPV, risk factor studies, and correlation studies (7, 23–27). But many perinatal women who suffer from IPV say they are not asked by obstetricians or midwives about intimate violence (28). This incongruity underscores the urgent need to re-evaluate support frameworks by critically analyzing lived experiences. A nuanced understanding of lived experiences is essential in informing effective, empathetic interventions. Qualitative research is particularly well-suited to capturing the Inner feelings and experiences of perinatal women suffering from IPV (29). And providing a contextualized framework for clinical practice. Meta-synthesis offers a valuable method for synthesizing qualitative evidence, allowing for a deeper exploration of complex emotional and psychological processes.

Therefore, this study aims to systematically integrate the real psychological experiences and needs of perinatal women who have experienced or are currently experiencing IPV. It seeks to answer the following questions: (1) What are the experiences and needs of perinatal women who experience intimate relationship violence? (2) What recommendations can be drawn from these studies to inform clinical practice, education, and future research to better support Perinatal women who experience intimate relationship violence?

## 2 Methods

### 2.1 Study design

This study is a meta integrated research on the real psychological experiences and needs of women who have experienced or are currently experiencing IPV during the perinatal period, and followed the guidelines for improving the transparency of qualitative research synthesis reports (ENTREQ) (30) for reporting.

### 2.2 Data sources

We retrieved qualitative studies on intimate relationship violence experienced by perinatal women from Chinese and English databases PubMed, Cochrane Library, Web of Science, CINAHL, PsycINFO, Embase, Ovid, as well as China National Knowledge Infrastructure, Wanfang Database, VIP Database, and China Biomedical Literature Service System using computer systems. The retrieval deadline is from the establishment of the database to July 2025. Searches were based on a combination of free-text keywords and indexed terms (MeSH) related to the terms: intimate partner violence, violence against women, domestic violence, pregnancy, perinatal, qualitative research etc. (The search strategy is shown in Supplementary File 1).

### 2.3 Eligibility criteria

According to PICoS, the inclusion criteria for research literature are as follows. Inclusion criteria: (1) The research subjects (population, P) are perinatal women, including family members or non-family members (friends or medical personnel) of perinatal women, and only the inner experiences, feelings, and coping strategies of perinatal women themselves are extracted; (2) The phenomenon of interest (I) refers to the true psychological experiences, feelings, needs, and coping strategies of pregnant women who have experienced or are currently experiencing intimate relationship violence throughout the entire perinatal period; (3) Context (Co) refers to women experiencing intimate relationship violence during the perinatal period; and (4) The research design (S) is qualitative research, including phenomenological research, narrative research, grounded theory, ethnography, and as well as the qualitative research part in mixed research. Exclusion criteria: (1) inability to retrieve full-text, incomplete data, and duplicate publications; (2) Non-Chinese and English literature; (3) Adopting mixed research methods but unable to separate qualitative data; and (4) Conference abstracts, dissertations. Data collection continued until feasible sample size was met.

### 2.4 Study selection and data extraction

Literature screening and data extraction were independently completed by two researchers, followed by cross checking. In case of any discrepancies, the decision was made by a third researcher. If a decision still cannot be reached, the research team will discuss the matter and reach a consensus before making a decision. Firstly, we used Endnote X9 software to remove duplicate literature, then read the title and abstract to exclude obviously unrelated literature. Finally, we read the entire text and included literature that was relevant to this study. The data extracted by two individuals using an Excel spreadsheet, including author, publication year, region, qualitative research methods, research subjects and number, interested phenomena, and main research results. Enter the main research results into Nvivo20 software for text content synthesis and integration.

### 2.5 Quality assessment

Two researchers conducted literature quality evaluations separately, and if the evaluation results were inconsistent, a third researcher made a decision. The qualitative research quality evaluation criteria published by the Australian JBI Evidence based Healthcare Center were adopted (31). The evaluation consists of 10 items, each evaluated as “yes,” “no,” “not applicable,” or “unclear.” The literature quality is divided into three levels: A, B, and C. Fully meet the evaluation criteria, with a low possibility of bias, rated as Class A; Partially meets the evaluation criteria, with a moderate possibility of deviation, classified as level B; Completely unsatisfied, with a high possibility of offset, classified as level C. The final inclusion of studies with quality evaluation levels of A and B.

### 2.6 Research team and reflexivity

The research team consisted of the four authors. The authors conceived this study. All four authors worked in maternity hospitals. The four authors have a relatively in-depth understanding of the physiology and psychology of perinatal women. And trained in systematic qualitative research. The researchers’ first language and sociocultural background differed from those of the study participants. To fully understand the context of the qualitative studies included in the research. The researchers completed the study under the guidance of team members who were proficient in English. Read repeatedly and understand the meaning of the participant’s words. Efforts were made to minimize bias caused by the researchers themselves. After repeated discussions, the research team reached a consensus on the research results.

### 2.7 Data analysis

This study adopted the Thomas and Hardens’ thematic synthesis method (32): (1) Repeatedly read, analyze, and interpret the meaning of relevant research results based on a full understanding of the philosophical ideas and methodology of qualitative research; (2) Combine and summarize similar results together to form a general category; and (3) Further categorize and form integrated concepts or explanations. Through these three steps, the reports incorporated into the literature were reported as a whole, and new core themes were distilled and given new interpretations. Two researchers independently coded and then aggregated the codes. In cases where there was disagreement, we consulted a third researcher for discussion and decision-making. The research team held multiple discussions on the final results until we reached a consensus. The details of the encoding formation process are shown in Table 1.

**Table 1:** Coding examples.

Quote	Codes	Sub-theme	Theme
I endured his behavior until one day he injured my finger… so severely that it deformed (42).	Physical violence	Aggravated physical discomfort symptoms	Negative experience
My husband is lying on top of me when he wants to have sex, but I’m pregnant and I feel a lot of pain… makes me very sad (21).	Sexual violence		
I always have a headache. I think too much, and whenever I think of my husband, I lose my appetite to eat (41).	Physical discomfort		
Even the hospital psychologist says that the high blood pressure crisis that I had may have developed due to the anger that I experienced during childbirth (33).	Adverse pregnancy outcomes		
I tell him am sick but he takes it easy like am not sick he ignores me without giving me money to go to the health facility… (21)	Neglect	Severe psychological trauma	
He does not care about me at all. But I’m pregnant, so I really need some words of encouragement… even if I speak to him gently, he will shout at me (41).			
He started telling me things, hurting me emotionally, telling me that I′ m a fool, and stupid, I′ m an idiot (20).	Verbal attacks/insults		
He(husband) criticizes me because I’ve gained weight after the birth. He says that I eat too much and that my skin is too dark (41).			
When I was playing social media, he immediately threw away my phone… he no longer allows me to use my phone (41).	Manipulation/control		
I want to go to work, and then he stopping me from going to work. Then he starts saying abusive words to me (43).			

## 3 Results

### 3.1 Study characteristics

**Figure 1:**
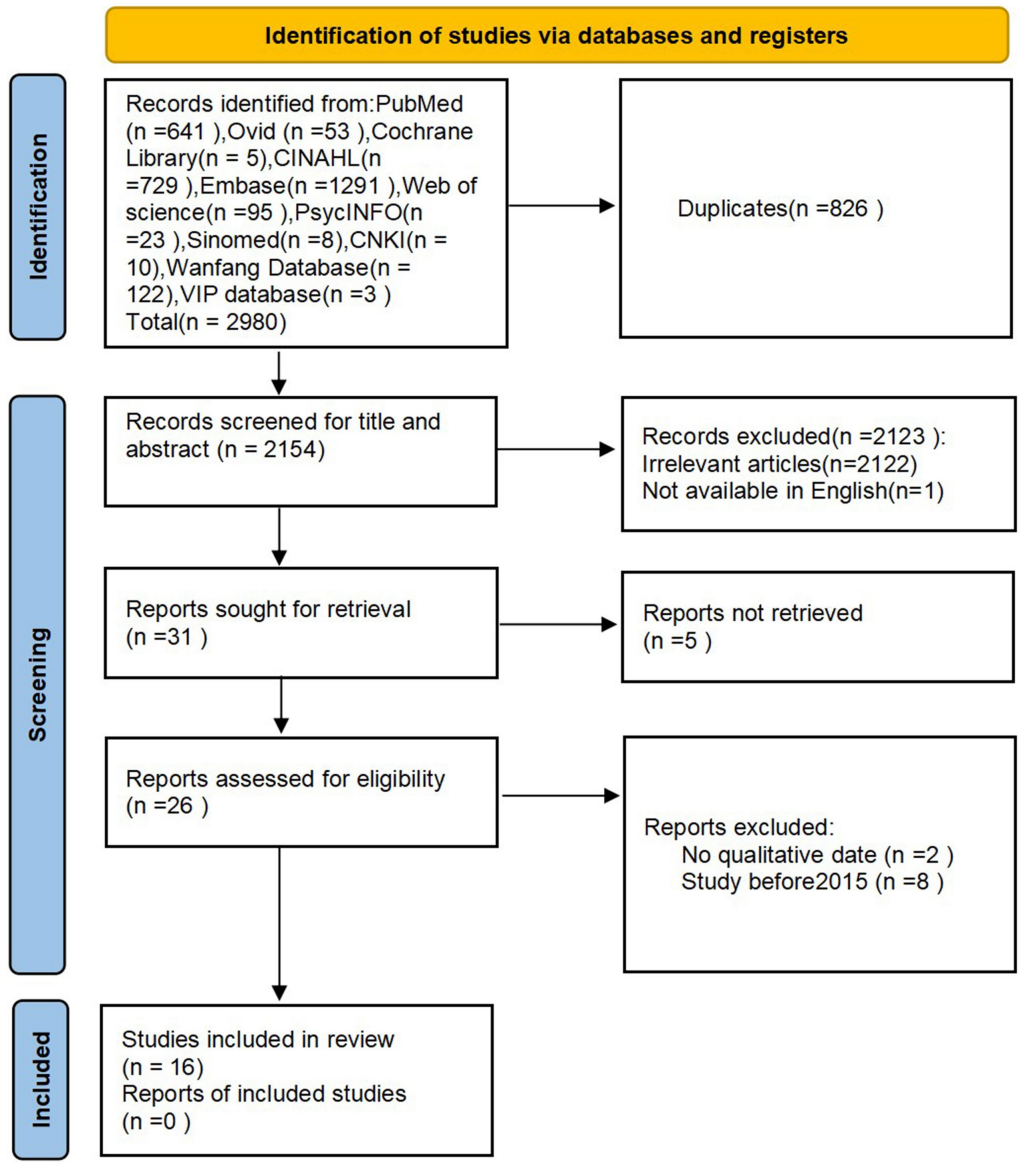
PRISMA flowchart.

A preliminary search obtained 2,980 Chinese and English literature, and after removing duplicate literature, 2,154 were obtained. By reading the literature titles and abstracts, 31 literatures were obtained after excluding literature unrelated to the topic and non-Chinese English literature; After reading the full text, 15 articles that cannot be obtained and had low quality (with a quality evaluation level of C) were excluded. Finally, 16 articles were included, and the literature screening process is shown in Figure 1. 16 articles are all in English (20–22, 28, 29, 33–43), and the basic characteristics of the included articles are shown in Table 2. All included studies were published between 2016 and 2024 and conducted in 13 different countries, with a total of 388 participants. Participants were recruited through purposive sampling or convenience sampling methods, and the interview locations involved in the study included prenatal care facilities, prenatal clinics, participants’ homes, or other private rooms. Three studies (33, 39, 41) adopted a mixed method design, in which only qualitative data was extracted. Other studies (20–22, 28, 29, 34–38, 40, 42, 43) are qualitative studies that use case analysis, content analysis, exploratory research, grounded theory, explanatory phenomenology, and descriptive phenomenology methods. Qualitative data was collected through semi-structured interviews.

**Table 2:** Summary of included studies.

Author	Sample	Age of participants	Aim(s)	Design	Main findings
Kirwan (29), Ireland	40 pregnant women; 30 medical personnel	20–39	Exploring the views of women and providers on IPV routine inquiry as a routine aspect of prenatal care	Case analysis and research	Safe Space. Education and awareness. Pregnancy—a unique opportunity. A right and a duty of care. Enabling preparedness in their trajectory toward safety.
Sánchez (33), Brazil	12 women receiving prenatal and postpartum care services (including women who have experienced domestic violence)	12–32	Explore women’s perceptions of violence in Brazilian society, its causes, manifestations, consequences, and strategies for preventing and responding to domestic violence against women.	Mixed research	Between the public and the private spheres—violence against women and its manifestations, causes and particularities. Forms of violence. The roots of violence against women. Cycle of violence: its peculiarities, consequences and the barriers to interruption. Factors that increase vulnerabilities: The female condition. Pregnancy and the postpartum period. The COVID-19 pandemic. History of family violence and socioeconomic inequities. Mental health and substance abuse. Protection system and support network—strengths and weaknesses. Alternatives for the prevention and elimination of violence.
Fernández (34), Spain	6 women who have experienced IPV during pregnancy	38–48	Determine the needs, concerns, and preferences of IPAW (Intimate Partner Violence Against Women) for the electronic health strategy.	Descriptive phenomenological research	Needs and concerns of pregnant women in an IPVAW situation. Support (Role of health care professionals, Psychological and emotional support). Concerns (Children and the unborn baby, Barriers for leaving the relationship). Video counseling intervention: key aspects. Contents (Awareness, Self-esteem and fears, Legal advice). Video-based counseling (Feasibility, Safety, Barriers). Contributions for safety planning apps.
Barez (35), Iran	13 women who suffered from domestic violence during the perinatal period, 1 husband and 1 daughter, and 24 key informants	19–41	Exploring strategies for Iranian women to deal with domestic violence during pregnancy	Qualitative study	Escape strategies: Concealment (Concealment of violence). Passive dysfunctional behaviors (Emotional release, Retaliatory behaviors, Abuse to husband and child, Helplessness and confusion, Recourse to divorce). Neutral behaviors (Placating strategies, Diverting attention). Situation improvement strategies: Active self-regulation (Self-actualization, Comprehensive self-care skills, Promoting positive self-concepts, Resilience, Strengthening spirituality). Protecting family privacy (Constructive purposeful efforts, Supportive efforts, Maintaining maternal commitment, Preserving marriage, Avoiding social judgments). Help seeking (Disclosure of violence, Looking for network support).
Herbell (36), America	11 women who have suffered from IPV currently or in the past year	18–24	Explore participants’ views on IPV experiences, parenting styles, and security strategies.	Grounded theory study	“Broken Spirit”. Making sense of abusive episodes. A sense of anger and resolve. “No choice but to deal with it.” I want better for my kids and me. Prioritizing the child’s needs. Parenting in the context of abuse. Safety planning as an element of parenting. Looking for “red flags.” Motivation for the safety plan: lack of trust in others. Elements of the safety plan.
Garnweidner-Hoime (22), Norway	8 pregnant women who experienced different types of IPV during pregnancy	-	Investigating the IPV experiences and recommendations of pregnant women in prenatal care in Norway	Exploratory research	Women’s experiences with communication about IPV during antenatal care. The midwife did not ask about violence. Antenatal care was a good arena. Lack of facilitators to talk about IPV. Midwives were perceived as powerless. Women’s advice on how to communicate about violence in antenatal care. Starting the conversation about IPV. Importance of explaining what violence is. Communicating about IPV toward the end of pregnancy. Organizing antenatal care. Providing helpful materials to talk about IPV. Facilitators to talk about IPV in antenatal care. The midwives’ knowledge about violence. Information about health consequences. The consequences of disclosing violence. The use of a professional interpreter. Barriers to talk about IPV in antenatal care. Being accompanied by the husband. Fear of the Child Welfare Service. Talking about violence not being accepted in their culture. Fear that nobody would believe them.
Rishal (37), Nepal	12 women who experienced domestic violence during pregnancy	22–45	Exploring how Nepalese women evaluate their prenatal care and their expectations regarding prenatal care in relation to domestic violence during pregnancy	Describe qualitative research	Enduring domestic violence a hidden burden. Concealment. Barriers to disclosure. All we need is an opportunity. Routine enquiry. Support focused on women. Other support for family members. Made a bad thing worse. Neglect. Emotional abuse. Physical abuse.
Lévesque (38), Canada	17 women who have experienced domestic violence at least once during their perinatal period in the past five years	Most age 30	Focus on the challenges faced by mothers in the context of domestic violence	Describe qualitative research	A Parental Experience Impoverished by the Context of DVPP, Caused by an Increase in Tasks, a Weakened Bond with the Child and a Loss of Self-Confidence. The Baby’s Arrival and the Father’s Lack of Investment in Parenthood Place an Additional Burden on the Mother. The Emotional Bond with the Child is compromised by the Violence. Psychological Violence Instilled lack of Confidence About Parenting Abilities. Parenting responsibility in a context of DVPP is expressed as greater vigilance and the need to protect the child from violence. Living in a Climate of Fear and Hypervigilance, for the Present and the Future. The Absence of a Safe Environment for the Child promotes the Deployment of Protection Strategies, Including Separation from the Abusive partner. The Ideal Family and Custody Issues as Obstacles to Separation. The Mother’s Social Circle Influenced the Decision to Remain or Leave. Parenting Practices: The Children as Targets and Victims of Violence, Resulting in Additional Child Needs That Mothers Must Meet. Children as a Vector of Violence and Control. Care contexts aggravated by violence.
Yirgu (39), Ethiopia	24 women who experienced any IPV during pregnancy	Age mean:26.8	During the COVID-19 pandemic, the needs and unmet needs of IPV survivors in terms of support services	Mixed research	Knowledge of IPV services. Experiences of women in seeking IPV services. Challenges to accessing IPV services. The impact of COVID-19 pandemic on access to and utilization of IPV services. Persistent service needs for women experiencing IPV.
Katushabe (21), Uganda	25 women who experienced any IPV during pregnancy	15–35	Exploring the IPV experience, coping strategies, and seeking support for pregnant women seeking treatment at a city hospital in southwestern Uganda.	Explanatory phenomenological research	IPV experiences. Psychological violence. Sexual violence. Physical violence. Economical violence. Lack of financial support. Women as sole family breadwinners. Deprived of gainful employment. Taking away all the money. Support seeking. Barriers to support seeking. Midwives’ involvement. IPV coping strategies among pregnant women. Confiding in relatives. Keeping Quiet. Praying to God. Disrupting negative thoughts. Reporting to authorities. Self-consolation. Partner tolerance. Man as a dominant force. State of resignation. Understanding spouse’s likes and dislikes. Saying yes to everything. Belief that all men are the same. Intergenerational continuity of IPV.
Barez (28), Iran	14 women who experienced domestic violence during pregnancy until one year postpartum	19–37	Exploring the Experience of Iranian Pregnant Women Suffering from IPV and Identifying Their Neglected Needs	Describe qualitative research	Family and society empowerment. Need to empower couples to reduce domestic violence during pregnancy (Need to enhance couples’ awareness, Necessity of improving couples’ life skills, Enabling women’s economic empowerment, Demand for preserving individual values). Demand for improved health care services (Health care system informational and skills empowerment, Professional ethics requirements, Service demand for psychological care, Need to maternity care improvement, Investing for health care infrastructures, Empowering the health education system). Need to strengthen inter sectoral, legal and social supports (Provision and strengthening inter-sectoral support, Reform the educational system, Reform and implementation of women’s protection laws, Need to receive social support).
Keynejad (40), Ethiopia	16 pregnant women; 12 healthcare workers	16–39	Views of rural pregnant women and healthcare workers in Ethiopia on perinatal mental health and intimate partner violence	Describe qualitative research	Theme 1: “He threatened to cut me into pieces” – intimate partner violence. Theme 2: “not… the end of the world if a husband beats his wife” – patriarchal norms. Theme 3: “She cannot do anything to protect herself”-powerlessness. Theme 4: “I do not always feel happy … I feel sick” – emotional and bodily distress.
Hatcher (20), South Africa	Women receiving routine prenatal care (13), pregnant women suffering from IPV (5), healthcare providers (8), health managers and researchers (10), non-governmental organizations (6), community leaders (4)	-	Exploring the views of patients, healthcare providers, and community members on evaluating and resolving IPV in this situation	Describe qualitative research	Awareness of IPV. Receptiveness to Intervention. Clinic Considerations for IPV Interventions. Toward a Feasible, Sustainable Intervention.
Nhi (41), Vietnam	20 pregnant women who have experienced emotional violence and developed depressive symptoms during pregnancy or after birth	17–37	Exploring how women living in northern Vietnam suffer emotional abuse from their husbands and how they assess the impact of such tense intimate relationships on their mental health	Mixed research	Being ignored. Being denied support. Being controlled. Consequences of emotional violence for women’s mental well-being.
Sigalla (42), Tanzania	18 women who experienced physical IPV during pregnancy	18–39	Exploring the Support Provided by Native Families to Women Suffering from IPV During Pregnancy	Describe qualitative research	Whether or not to seek support. Severity of violence. Feelings of inferiority. Doubts about the natal family’s capacity to help. Social support provided by the natal family. Emotional support. Information/Advice. Practical support. Mediation.
Scott (43), America	20 women who suffered economic violence during the perinatal period; 18 staff members engaged in handling domestic violence related matters	18–64	(1) The manifestations of economic abuse during pregnancy and the perinatal period; (2) The impact of economic abuse on women during the perinatal period; (3) How to effectively support perinatal women who have suffered economic abuse.	Describe qualitative research	Domain 1: Individual experiences of economic abuse. Theme 1: Survivors and advocates described a wide range of experiences of economic abuse during the perinatal period. Theme 2: Impacts of perinatal economic abuse on health, finances, and access to necessities. Theme 3: Survivors emphasized economic abuse as a barrier to leaving their relationship. Domain 2: Using systems to harm survivors. Theme 4: Partners used child welfare, legal, and public benefit systems to financially harm survivors. Theme 5: Partners used survivors’ intersectional identities to substantiate and exacerbate economic abuse during the perinatal period. Domain 3: Needed resources & support for perinatal & parenting survivors of economic abuse. Theme 6: Additional resources, such as education on economic abuse, supportive employment policies, and direct cash assistance, are needed to support survivors’ economic thriving during the perinatal period.

### 3.2 Quality assessment results

The quality evaluation of five articles was rated as A (20, 28, 29, 38, 40), and the quality evaluation of eleven articles was rated as B (21, 22, 33–37, 39, 41–43). Fifteen studies did not mention whether the philosophical foundation and methodology were consistent; Eleven studies (21, 22, 33–37, 39, 41–43) did not explain the researchers’ own situation from the perspective of cultural background and values; Twelve studies (21, 22, 33–39, 41–43) did not elaborate on the impact of researchers on the research or the influence of research on researchers. Of the sixteen studies, five studies were from Africa (20, 21, 39, 40, 42), four studies from Asia (28, 35, 37, 41), three studies from Europe (22, 29, 34), three studies from North America (36, 38, 43) and one study from South America (33). The results of the literature quality evaluation are shown in Table 3.

**Table 3:** Results of quality assessment.

Author	1	2	3	4	5	6	7	8	9	10	Overall rating
Kirwan	Unclear	Yes	Yes	Yes	Yes	Yes	Yes	Yes	Yes	Yes	A
Sánchez	Unclear	Yes	Yes	Yes	Yes	No	Unclear	Yes	Yes	Yes	B
Fernández	Unclear	Yes	Yes	Yes	Yes	No	No	Yes	Yes	Yes	B
Barez	Unclear	Yes	Yes	Yes	Yes	No	Unclear	Yes	Yes	Yes	B
Herbell	Unclear	Yes	Yes	Yes	Yes	Unclear	Unclear	Yes	Yes	Yes	B
Garnweidner-Hoime	Unclear	Yes	Yes	Yes	Yes	No	No	Yes	Yes	Yes	B
Rishal	Unclear	Yes	Yes	Yes	Yes	No	Unclear	Yes	Yes	Yes	B
Lévesque	Unclear	Yes	Yes	Yes	Yes	Yes	Unclear	Yes	Yes	Yes	A
Yirgu	Unclear	Yes	Yes	Yes	Yes	No	No	Yes	Yes	Yes	B
Katushabe	Unclear	Yes	Yes	Yes	Yes	No	No	Yes	Yes	Yes	B
Barez	Unclear	Yes	Yes	Yes	Yes	Yes	Yes	Yes	Yes	Yes	A
Keynejad	Unclear	Yes	Yes	Yes	Yes	Yes	Yes	Yes	Yes	Yes	A
Hatcher	Unclear	Yes	Yes	Yes	Yes	Yes	Yes	Yes	Yes	Yes	A
Nhi	Unclear	Yes	Yes	Yes	Yes	No	No	Yes	Yes	Yes	B
Sigalla	Unclear	Yes	Yes	Yes	Yes	No	No	Yes	Yes	Yes	B
Scott	Unclear	Yes	Yes	Yes	Yes	No	No	Yes	Yes	Yes	B

### 3.3 Key findings of the meta-synthesis

**Figure 2:**
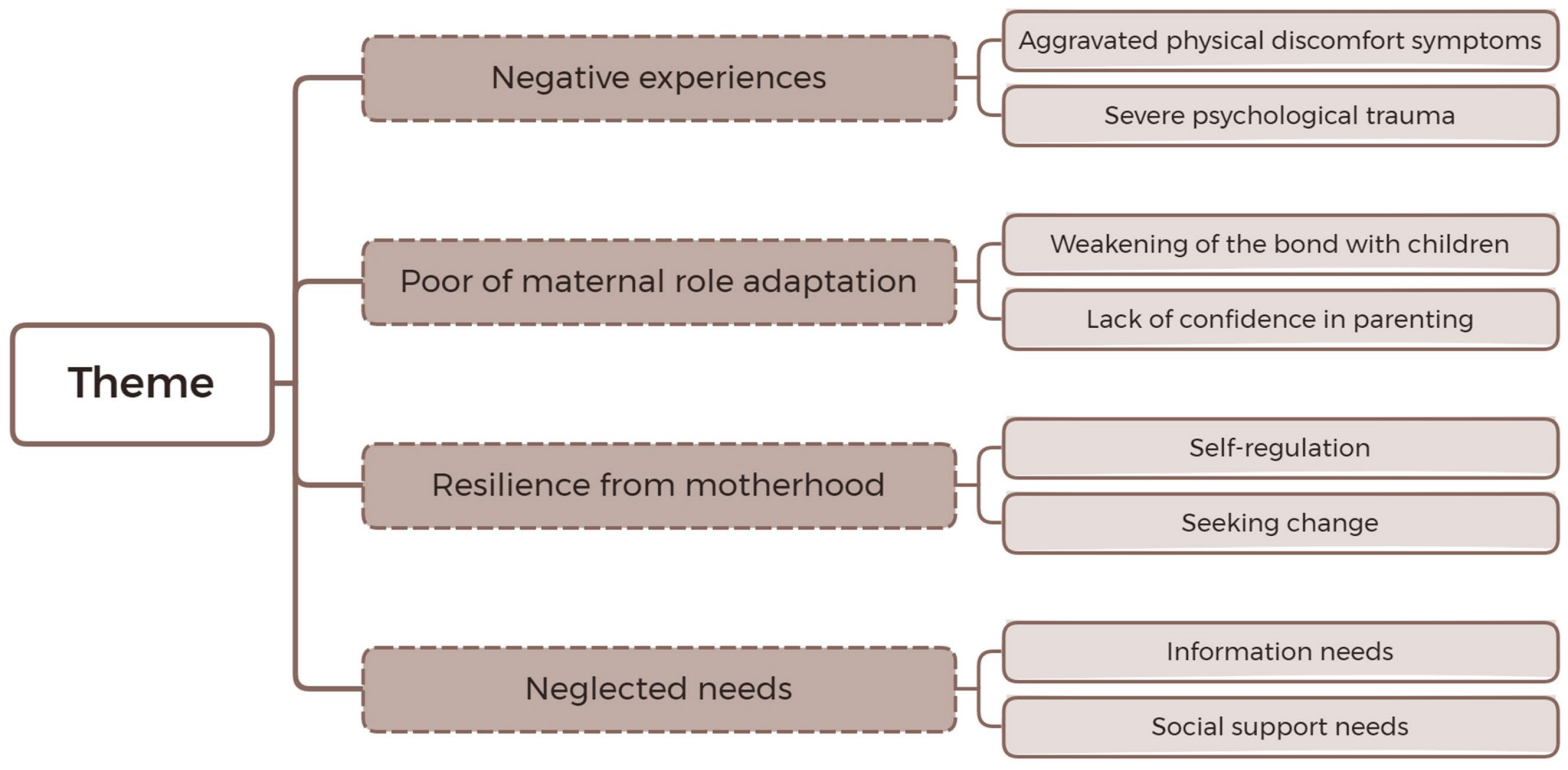
Theme flowchart.

Thematic analysis of 56 key quotes from the 16 included studies generated four overarching themes: Theme 1: Negative experiences (① aggravated physical discomfort symptoms, ② severe psychological trauma); Theme 2: Poor of maternal role adaptation (① Weakening of the bond with children, ② Lack of confidence in parenting), Theme 3: Resilience from motherhood (① Self-regulation, ② Seeking change); Theme 4: Neglected needs (① Information needs, ② Social support needs). A chart was created to summarize the main themes and their interrelationships (Figure 2).

### 3.4 Qualitative meta-synthesis

#### 3.4.1 Negative experience

##### 3.4.1.1 Aggravated physical discomfort symptoms

There were various forms of IPV, among which the most serious and harmful was physical violence, which caused irreversible physical and mental trauma to women during the perinatal period (33, 37). Forms of physical violence include slapping, pushing, pulling, kicking, stepping, etc. (21), which leaved serious bruises on women and pose a serious threat to the safety of women and/or fetuses (42, 44). Sexual violence was also one of the important reasons for exacerbating women’s physical discomfort (21). The decrease in women’s willingness to engage in sexual activity during the perinatal period made it a high-risk period for sexual violence (21). Some women expressed that they were often forced or uncomfortable to have sexual intercourse with their husbands (21). Due to long-term stress, many perinatal women who suffered from IPV experience varying degrees of physical discomfort symptoms, such as headaches, insufficient or excessive sleep, nightmares, and decreased attention (40, 41). Physical violence leads to serious adverse pregnancy outcomes such as miscarriage, complications of pregnancy, fetal loss, and maternal deaths (20).

“My husband is lying on top of me when he wants to have sex, but I’m pregnant and I feel a lot of pain… makes me very sad (21).” “I always have a headache. I think too much, and whenever I think of my husband, I lose my appetite to eat (41).”

##### 3.4.1.2 Severe psychological trauma

Emotional violence such as verbal attacks, indifference, manipulation, and control was more common and covert than physical violence, causing serious psychological trauma to women in the perinatal period (33, 37). Some women have expressed that their needs have been ignored (21, 41). Some women have reported experiencing neglect and verbal attacks from their intimate partners (20, 41). Some women expressed that their freedom was restricted (41). The husband controls their social activities, clothing choices, movements and finances (21, 40, 43). Their husbands monitor their phones and restrict their travel, this placed the women in a situation of existential isolation (41). They want to have social relationships with friends or relatives (41). Neglect, control and verbal insults caused long-lasting psychological trauma to women in the perinatal period, increases risk of perinatal depression (43, 45).

“I started crying after I got pregnant because my husband was always angry or scolding me, maybe I had depression (41).” “When I was playing social media, he immediately threw away my phone… he no longer allows me to use my phone (41).” “I want to go to work, and then he stopping me from going to work. Then he starts saying abusive words to me (43).”

#### 3.4.2 Poor of maternal role adaptation

##### 3.4.2.1 Weakening of the bond with children

Experiencing IPV during the perinatal period can lead to role transition disorders (36, 38). This makes it difficult to adapt to the role of a mother. It is not conducive to the formation of a good mother child attachment relationship (38). Some women expressed that the bond between themselves and their children has weakened (37). Some women believe that children are the result of their husbands’ violence (35). And because they detest their husbands, they also detest their children. Even by punishing the children to vent one’s dissatisfaction (35). As a result, they are unable to establish an emotional connection with their children (38).

“For me, he represented the domestic violence. The product of… a bad relationship. It was… wow. No, I did not love that child (38).” “When I was angry because of my husband’s violent behavior, I hit the baby in my stomach and empty myself in such a way (35).”

##### 3.4.2.2 Lack of confidence in parenting

Some women frequently suffer from violence from their husbands (29). They are unable to fulfill their responsibilities as mothers (29, 36, 38). They cannot properly take care of their children (40). They lack the knowledge and skills for parenting, and ultimately lose confidence in parenting (38, 40). Some women expressed great guilt about their children (36). Some women have reported that their partners use their children to attack, manipulate, and threaten them (35). These dilemma all led to their inability to fully engage in their new roles, interact with children, and fulfill their responsibilities of raising children (38).

“I’m trying not to let my insecurities rule my parenting (36).” “After the birth, I was constantly doubting myself as a mother… Maybe I’m not doing enough? … I lost my bearings. Because him, he was constantly criticizing me (38).” “He (husband) now knows that the best way he can hurt me, attack me, or retaliate against me is through his children (35).”

#### 3.4.3 Resilience from motherhood

##### 3.4.3.1 Self-regulation

Perinatal women exposed to IPV apply situation improvement strategies to protect themselves and their children (35, 38). Some women choose to improve their situation through self-regulation. Self-regulation through self-actualization, comprehensive self-care skills, promoting positive self-concepts, resilience and strengthening spirituality (21, 35). And creating a good mood, self-relaxation, return attention through enjoyable activities, positive mental imagery and maintain authority, skills and empowerment (21, 35, 40). Maintaining and promoting self-confidence, self-esteem and self-control (35).

“I tried to calm down by shifting my focus, thinking about myself and the child in my belly, which made me stop thinking about violent things (35).” “Some pregnant women lost their self-confidence during pregnancy…I always told I’m very good and I have no problems (35).”

##### 3.4.3.2 Seeking change

Out of the mother’s instinct to protect the fetus or the newborn. Women who have suffered from long-term IPV during the perinatal period are eager to change this undesirable situation (35, 36, 38). Some women ultimately chose to end unhealthy intimate relationships (33, 34, 36). Some women said they fought back against their husbands’ abuse and sought help from their families of origin (21).

“I should make a decision (to leave my husband) because I do not want my daughter to live in such an environment after giving birth (33).” “When my husband abused me, I retaliated against him. He wanted to hit me in the abdomen, so I fought back in self-defense (35).” “My father once said: Daughter, please leave him (your partner), we will take care of these children and send them to school (42).”

#### 3.4.4 Neglected needs

##### 3.4.4.1 Information needs

IPV related knowledge: Most women who suffered from IPV during the perinatal period expressed a lack of knowledge about IPV (20–22, 29, 39). They were eager to learn about it in order to identify and seek help as early as possible (20, 28, 35). When seeking help through online or mobile app platforms, it is necessary to ensure sufficient security and privacy during use (34). Legal and psychological counseling information: Perinatal women who had experienced intimate relationship violence hoped to receive legal and psychological support (21, 28, 34, 43). They also want to understand issues such as divorce, property division, and custody (34, 43). Meanwhile, the respondents hoped that society can provide them with multilingual legal publicity services (43).

“I wish I could know that not having financial support is also a form of abuse (43).” “We should have a flowchart… with a route for seeking help (22).” “I want to know how to get a divorce and about custody issues (34).” “I do not know if the health center has a psychologist who provides free counseling for me. I need psychological counseling (28).” “Abused mothers should be informed about support systems. What services can they receive from the health centers? Where can they go for psychological counseling? (35).”

##### 3.4.4.2 Social support needs

Most informal social support system support from family and friends (21, 42). Perinatal women who have suffered from IPV long for support and assistance from the trustworthy people around them (42, 43). The support of family members, such as providing emotional support, advice, and practical assistance, can help them avoid the harm caused by violence (42). Formal social support system support from the health care system, welfare organization, social emergency, social work, forensic medicine, judicial and legal system, and police (28, 43). Some of the participants recognized the causes of violence in historical, cultural and structural elements (33). Therefore, a formal institution is needed to carry out strong intervention, such as taking measures to get women back into the job market (21, 28, 43). Or introduce policies aimed at changing the public’s perception of gender inequality (28, 33). Some women during the perinatal period craved support and assistance from professional medical institutions (43). Many women expressed that they hoped healthcare professionals can take their pain seriously and understand it (34).

“He (father) asked me to go home because he still loves me very much (42). “They (medical staff) should ask us what happened… who abused us (37).” “There should be a 24- hour telephone counseling center so that they can call for psychological advice (28),” “Society should react to violence against pregnant women… (28).” “The woman must be the person who takes care of the children and does not have the kind of support network that allows her to return to the job market, right? It’s always the woman who ends up giving up (33).”

## 4 Discussion

This study systematically synthesized 16 qualitative articles to explore the experiences and needs of perinatal women who have experienced or are currently experiencing IPV. Through coding, constant comparison, and analysis, these experiences were organized into four themes (Negative experiences, poor of maternal role adaptation, resilience from motherhood; and neglected needs).

The respondents in this study came from countries in Europe, Asia, Africa, North America and South America. This indicates that intimate relationship violence is widespread in most parts of the world. The research focuses on the countries in Africa and Asia (20, 21, 28, 35, 37, 39–42). This is consistent with the research results of Jean et al. (46). The rate of IPV is the highest in the Eastern Mediterranean region (46). Social and cultural factors have contributed to the increase in the incidence rate in this region. The countries in this region share many common social and cultural characteristics, including the “silence” surrounding IPV. Brunelli et al. (3) indicates that physical violence and sexual violence occur more frequently in Africa and the Middle East. Psychological violence is more common in the Americas and the Eastern Mediterranean region (3). Countries with higher incomes have a lower rate of violence (3). The research conducted by Chen et al. (47) indicate IPV prevalence in urban slums was notably high. Tran et al.’s (48) study also pointed out that young people from low-income families, those living in rural areas, and those with low educational attainment tend to be more tolerant of IPV.

Gender inequality resulting from factors such as race, history, economy, culture and social structure is an important influencing factor for perinatal women to suffer from IPV (33). And the constraints of traditional culture, religious beliefs, imperfect social systems, the fear of exposing the consequences of IPV, and the economic and emotional dependence on the abusers… all these complex factors have led them to choose to remain silent about IPV (33, 37, 41, 42). At the same time, the issue of basic sustenance makes it even more difficult for women to escape the predicament of IPV (20, 40). This is consistent with Park’s research (49) findings. Improving perceptions of gender inequality (at the individual level) and emphasizing democratic values (at the national level) are of great significance in reducing intimate relationship violence.

Intimate relationship violence encompasses physical, psychological, sexual and economic violence. It causes severe trauma to the physical and mental health of pregnant women during the perinatal period. Moreover, it leads to serious adverse pregnancy outcomes (21). Perinatal women who have suffered from IPV have demonstrated remarkable resilience and recovery ability in order to protect their children. Most women choose to endure IPV in an attempt to preserve the integrity of their families, which is consistent with the research findings of Gilliam et al. (50). The frequent occurrence and intensification of violence forced them to adopt other coping methods. For instance, diverting attention, praying to God, and enhancing spirituality can enable mothers to remain calm when dealing with domestic violence (21, 35). Some other women, in the end, chose to divorce their partners in order to protect their children. Most of these women are educated, financially capable, and have family members providing support (42).

IPV has affected the parenting experiences and responsibilities of women during the perinatal period. Their violent partners used a variety of strategies to attack the mother’s parenting practices. For example by undermining her authority, interrupting her sleep as well as the baby’s, using the children to manipulate her, abusing the children directly, and more (38, 51). The crying or screaming of the children can bring back traumatic memories of violence for them. This has dampened their confidence in parenting and made them unable to fully focus on taking care of their children (38). This is consistent with the research results of Chiesa et al. (52), IPV victimization diminished levels of communication, connectedness, lack of effective parenting skills, as well as higher levels of physical aggression, neglect and authoritarian parenting styles. Living in an environment of IPV for a long time can easily lead to the intergenerational transmission of violence. Children tend to imitate their fathers’ behaviors in conflict situations, which seriously affects their growth (53). Therefore, it is necessary to assist pregnant and postpartum women in ending IPV, while also providing them with parenting knowledge and skills assistance.

Perinatal women who have suffered from IPV encounter significant obstacles when seeking help. Many women lack awareness of the forms of mental violence, economic violence and sexual violence. They are not aware that they have suffered IPV (22). Firstly, relevant knowledge about IPV should be provided to pregnant and postpartum women. They should be made aware that they have suffered from IPV. And they should take immediate measures and seek help to protect themselves and their children. Secondly, provide training on violence prevention and perinatal health care knowledge to the spouses. This helps men enhance their understanding of pregnancy-related matters (28). Studies (21, 39) have shown that the majority of IPV survivors rely on their informal support networks, such as family, friends, acquaintances, relatives, neighbors, etc. Raise public awareness of IPV. This can enable members of informal support organizations to recognize the dangers of IPV. Provide assistance to women and children.

The World Health Organization proposed that routine questioning of IPV was a right of perinatal women, a responsibility of healthcare providers, and a part of safe and complete routine prenatal care (29). Develop intervention guidelines for healthcare professionals to identify and manage perinatal IPV and eliminate violent behavior (35). Midwives should establish a more trusting and close relationship with pregnant women during the perinatal period (29). Their cultural values and language should be respected. Cultural security is a factor that determines whether they are willing to disclose information about IPV (54). At the same time, they should ensure safety and privacy during the conversation (37). Healthcare personnel can use simple questionnaire surveys or euphemistic questions such as “How have you been living recently?” and “What happens when you have different opinions with your partner?” as entry points to inquire about the sensitive topic of IPV (34, 55). Consultation via a questionnaire from an obstetric clinic or video on an online platform is acceptable. For example, video counseling is provided through e-health, and some “quick exit” buttons are set to ensure that women can use it safely (34). Provide written information materials related to IPV for women in the perinatal period, including response procedures and contact information for supporting organizations, such as placing written materials on bulletin boards in hospitals or health service centers (22).

Women who suffered from IPV during the perinatal period often bore significant physical and mental pressure for a long time. Taghizadeh (56) offered “problem-solving” skills training courses for women suffering from IPV at health centers, taught by systematically trained midwives on the best ways to cope with IPV. Can establish peer support groups to alleviate the loneliness of women suffering from IPV during the perinatal period by sharing stories from other women (34). Mercier et al. (57) systematically reviewed intervention measures for intimate relationship violence experienced by perinatal women, including cash assistance, family visits, counseling and education. Provide life skills training for perinatal women who have suffered from IPV (58). This can reduce IPV and improve the quality of the relationship. The specific training contents including conflict management, effective communication, anger management, problem-solving, emotional regulation, and stress management. Enhancing spirituality can enhance maternal–infant attachment behaviors (59). It helps protect mothers from the negative impacts of IPV.

Social formal organizations should also be aware of the dangers of IPV. The healthcare system, welfare organizations, forensic medicine, social work and legal institutions should collaborate together (28). Provide personalized and implementable assistance to women. For instance, providing employment, housing, child-rearing, and insurance-related policies. Offering cash assistance, training programs and education. Help women to end unhealthy relationships with their partners as soon as possible. Provide psychological counseling services. Conduct training on dealing with violence to enhance women’s safety (28, 39).

## 5 Limitations

Some limitations in our study should be considered. The inclusion of only English and Chinese publications may have introduced language bias. Additionally, the relatively small number of studies included (*n* = 16) may also reflect publication bias or gaps in qualitative research on Perinatal IPV in different regions contexts. Most studies do not mention the race, religion, and economic status of respondents, so there may be cultural differences in the results of this study.

## 6 Conclusion

This meta-analysis provides a systematic review of the psychological experiences and needs of perinatal women who experience intimate relationship violence, revealing that they suffer from severe psychological and physiological trauma Unable to adapt to the role of a mother, she longed to change the situation in order to provide protection for her child. And crave a comprehensive social support network to provide support and assistance. Perinatal IPV screening should be included in routine maternal and child health services, and healthcare workers need to receive training to remain sensitive and alert to issues that may be related to IPV. Provide them with timely psychological counseling and referral services. Strengthen the publicity and education on IPV, and enhance public awareness of this issue. The social support system should provide assistance to women who have experienced IPV during the perinatal period from multiple aspects such as economy, policies, laws and regulations, and psychological support. In the future, qualitative studies with large samples, multi-centers and trans-regions should be carried out. To explore the influence of different culture, race and economic level on perinatal IPV. This in turn provides more targeted clinical interventions.

## Data Availability

The original contributions presented in the study are included in the article/Supplementary material, further inquiries can be directed to the corresponding author.
