# Supplementary material for: Psychological experiences and needs of perinatal women experiencing intimate partner violence: a qualitative meta-synthesis
Source: Front Public Health. 2025 Oct 16;13:1678360. doi: 10.3389/fpubh.2025.1678360 (PMC12571652; doi:10.3389/fpubh.2025.1678360)
Supplement: Supplementary file 1 [file Table_1.docx]

**Supplementary 1.example search PubMed**

| #1 | ((((((pregnancy[MeSH Major Topic]) OR (Perinatal[Title/Abstract])) OR (Antenatal[Title/Abstract])) OR (postnatal[Title/Abstract])) OR (Breast feeding[Title/Abstract])) OR (lactation[Title/Abstract])) OR (childbirth[Title/Abstract]) |
| --- | --- |
| #2 | ((((((intimate partner violence[MeSH Major Topic]) OR (Violence against women[Title/Abstract])) OR (Domestic violence[Title/Abstract])) OR (Sexual violence[Title/Abstract])) OR (sexual abuse[Title/Abstract])) OR (verbal violence[Title/Abstract])) OR (physical violence[Title/Abstract]) |
| #3 | (((((qualitative research[MeSH Major Topic]) OR (qualitative study[Title/Abstract])) OR (phenomenology[Title/Abstract])) OR (experience[Title/Abstract])) OR (descriptive*[Title/Abstract])) OR (interview[Title/Abstract]) |
| #4 | ((((((((pregnancy[MeSH Major Topic]) OR (Perinatal[Title/Abstract])) OR (Antenatal[Title/Abstract])) OR (postnatal[Title/Abstract])) OR (Breast feeding[Title/Abstract])) OR (lactation[Title/Abstract])) OR (childbirth[Title/Abstract])) AND (((((((intimate partner violence[MeSH Major Topic]) OR (Violence against women[Title/Abstract])) OR (Domestic violence[Title/Abstract])) OR (Sexual violence[Title/Abstract])) OR (sexual abuse[Title/Abstract])) OR (verbal violence[Title/Abstract])) OR (physical violence[Title/Abstract]))) AND ((((((qualitative research[MeSH Major Topic]) OR (qualitative study[Title/Abstract])) OR (phenomenology[Title/Abstract])) OR (experience[Title/Abstract])) OR (descriptive*[Title/Abstract])) OR (interview[Title/Abstract])) |
